# Aberrant expression of microRNA-4443 (miR-4443) in human diseases

**DOI:** 10.1080/21655979.2022.2109807

**Published:** 2022-10-17

**Authors:** Yunan Mao, Jinze Shen, Yuchen Wu, Ruan Wenjing, Feng Zhu, Shiwei Duan

**Affiliations:** a Department of Clinical Medicine, Zhejiang University City College School of Medicine, Hangzhou, China; b Department of Clinical Medicine, the First School of Medicine, Wenzhou Medical University, Wenzhou 325035, China; c Sir Run Run Shaw Hospital, College of Medicine, Zhejiang University, Hangzhou 310016, China

**Keywords:** miR-4443, expression, cancer, target gene, signaling pathway

## Abstract

miRNA is a small endogenous RNA and an important regulator of gene expression. miR-4443 is abnormally expressed in 12 diseases including cancer. The expression of miR-4443 is regulated by 3 upstream factors. miR-4443 has 12 downstream target genes. miR-4443 inhibits the expression of its target genes, thereby affecting the migration, proliferation, and invasion of pathological cells. miR-4443 participates in 4 signaling pathways and plays a role in the occurrence and development of several diseases. In addition, miR-4443 can also promote resistance to multiple drugs. Here, this article summarizes the aberrant expression of miR-4443 and its pathogenic molecular mechanisms in human diseases, which provides clues and directions for the follow-up research of miR-4443.

## Highlights

- Dysregulation of miR-4443 affects the behaviors of diseased cells.
- miR-4443 is involved in the regulation of four signaling pathways.
- miR-4443 is associated with reduced drug sensitivity in cancer patients.

## Graphical Abstract

**Figure:**
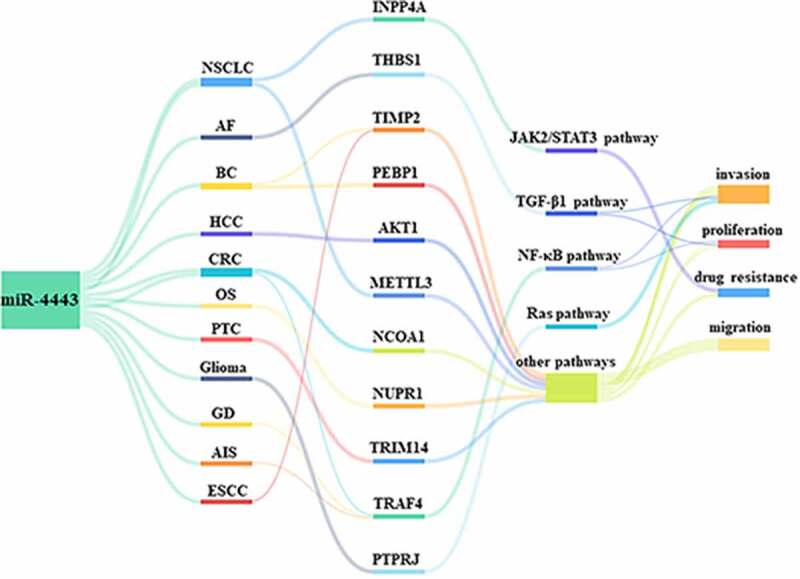

## Introduction

MicroRNA (miRNA) is a small non-coding RNA that can target and inhibit messenger RNA (mRNA) expression. miRNAs act similarly to other RNAs of the ribonucleoprotein (RNP) complex, providing sequence-specific binding components that enable RNPs to act on specific targets[1]. miRNAs typically bind to the 3’-UTR (untranslated region) of target mRNAs and inhibit protein production by degrading mRNAs and silencing translation [2].

In recent years, numerous studies have found that long non-coding RNAs (lncRNAs) may act as endogenous sponges to regulate the expression and function of miRNAs [3]. The competing endogenous RNA (ceRNA) axis is the main mode of action of lncRNAs [4]. Competing endogenous RNAs (ceRNAs) compete with common microRNAs (miRNAs) for binding, thereby inhibiting the targeting of downstream mRNAs by miRNAs [5].

There is increasing evidence that miR-4443, a recently discovered miRNA, is an important endogenous regulatory molecule. However, miR-4443 has not yet been comprehensively summarized. In this review, we outline the aberrant expression of miR-4443 in various diseases and its physiological significance. In addition, the relationship between abnormality of miR-4443 and activation of signaling pathway and drug sensitivity is also discussed. Our comprehensive overview of miR-4443 may provide potential clues for future studies of miR-4443.

## 1. Abnormal expression of miR-4443 in different diseases

### 1.1 Research progress of miR-4443 related studies

Previous studies have shown that miR-4443 is abnormally expressed in 9 cancers (Table 1). Among them, the samples or cell lines involved in miR-4443 research can be seen in Table 2. The up-regulation of miR-4443 in breast tissue is significantly associated with the risk of breast cancer (BC) and the invasion of breast cancer cells [6]. Up-regulation of miR-4443 expression was also associated with the risk of non-small cell lung cancer (NSCLC) [7] and Glioma [8]. In contrast, the down-regulation of miR-4443 expression in papillary thyroid carcinoma (PTC) cell lines is significantly related to the energy metabolism and metastasis of PTC in vitro [9]. In addition, the down-regulated expression of miR-4443 is associated with the risk of colorectal cancer (CRC) [10], osteosarcoma (OS) [11], hepatocellular carcinoma (HCC) [12], glioblastoma (GBM) [13], and head and neck squamous cell carcinoma (HNSCC) [14]. There are highly expressed ceRNAs of miR-4443 in these 4 tumors. These ceRNAs can competitively inhibit the expression of miR-4443, comprising lncRNA FEZF1-AS1 in OS and HCC, lncRNA MNX1-AS1 in GBM, and lncRNA LINC00460 in head and neck squamous cell carcinoma (HNSCC). In addition, miR-4443 was abnormally expressed in 3 non-tumor diseases. Up-regulation of miR-4443 expression in plasma is associated with the risk of acute ischemic stroke (AIS) [15], while down-regulation of miR-4443 expression in plasma is a risk factor of atrial fibrillation (AF) [16]. In CD4^+^ T cells, the significantly up-regulated expression of miR-4443 is also associated with the risk of Graves’ disease (GD) [17].

**Table 1.** The aberrant expression of miR-4443 and its signaling axis in human diseases.

Type	Expression of miR-4443	Level	Signaling axis	References
BC	upregulation	tissue and cell	miR-4443/PEBP1	[6]
	upregulation	tissue and cell	miR-4443/TIMP2/MMP2	[20,33]
HCC	downregulation	tissue and cell	FEZF1-AS1/miR-4443/AKT1	[12,21]
NSCLC	/	tissue and cell	miR-4443/METTL3	[36]
	upregulation	cell	miR-4443/INPP4A	[7]
CRC	downregulation	cell	miR-4443/NCOA1 and TRAF4	[10]
GD	upregulation	tissue and cell	miR-4443/TRAF4	[17]
OS	downregulation	tissue and cell	miR-4443/NUPR1	[11]
HNSCC	downregulation	tissue and cell	LINC00460/miR-4443	[14]
AIS	upregulation	cell	miR-4443/TRAF4	[15]
AF	downregulation	tissue and cell	miR-4443/THBS1	[16]
PTC	downregulation	cell	miR-4443/TRIM14	[9]
GBM	downregulation	tissue and cell	MNX1-AS1/miR-4443	[13]
Glioma	upregulation	cell	miR-4443/PTPRJ	[8]

miR-4443 is abnormally expressed in different diseased tissues or cells, thereby playing an important role in inhibiting target genes or participating in ceRNA regulatory axis.

BC, Breast cancer; HCC, Hepatocellular carcinoma; NSCLC, Non-small cell lung cancer; CRC, Colorectal cancer; GD, Graves’ disease; OS, Osteosarcoma; GBM, Glioblastoma; AIS, Acute ischemic stroke; PTC, Papillary thyroid carcinoma; AF, Atrial fibrillation; HNSCC, Head and neck squamous cell carcinoma;

**Table 2.** Samples and cell lines with aberrant expression of miR-4443.

Type	Sample	Expression of miR-4443	References
BC	Parental MDA-MB-231 and Epi resistant BC subline MDA-MB-231/Epi	higher in MDA-MB-231/ Epi	[33]
	BC cell lines MCF‐7, MDA‐MB‐231; human breast cell line MCF‐10A and normal human liver cell line THLE‐3; 3 BC tissues and normal tissues	higher in MCF-7	[6,20]
CRC	CRC cell lines DLD-1, HT-29, and HCT-116	lower in HCT-116	[10]
OS	58 OS samples and the morphologically nontumor tissues	lower in OS	[11]
HNSCC	HNSCC cell lines CAL-27, WSU-HN4, WSUHN6; 15 HNSCC tissues and paired normal tissues	lower in HNSCC	[14]
GD	40 GD tissues and 30 normal tissues	higher in GD	[17]
GBM	44 GBM samples and adjacent normal tissues	lower in GBM	[13]
Glioma	Astrocytoma cells U251-MG, U251	higher in Glioma	[8]
NSCLC	NSCLC EPI-resistant cell line H1299	higher in H1299	[7]
HCC	58 tumor-adjacent tissues and 58 HCC tissues	lower in HCC	[12]
PTC	30 TN and 30 PTC tissues; PTC cell lines TT, TPC-1, FTC-133, SW1736, MZ-CRC-1 and human thyroid epithelial cell line HTori-3	lower in PTC	[9]
AF	123 persistent AF patients and 100 healthy controls	lower in AF	[16]
AIS	108 AIS patients and 74 healthy controls plasma samples	higher in AIS	[15]

miR-4443 was highly expressed in BC, GD, Glioma, NSCLC, and AIS, while miR-4443 was low expressed in OS, HNSCC, GBM, HCC, PTC, and AF.

BC, Breast cancer; HCC, Hepatocellular carcinoma; NSCLC, Non-small cell lung cancer; CRC, Colorectal cancer; GD, Graves’ disease; OS, Osteosarcoma; GBM, Glioblastoma; AIS, Acute ischemic stroke; PTC, Papillary thyroid carcinoma; AF, Atrial fibrillation; HNSCC, Head and neck squamous cell carcinoma; Epi: Epirubicin.

Therefore, abnormal expression of miR-4443 affects the risk of various diseases. Competitive endogenous RNAs (ceRNAs) regulate genes at the post-transcriptional level by competing for miRNAs [18]. In 4 types of tumors, there are highly expressed ceRNAs that regulate cancer risk by inhibiting miR-4443 expression.

### 1.2 Pan-cancer analysis of miR-4443 using TCGA database

**Figure 1.**
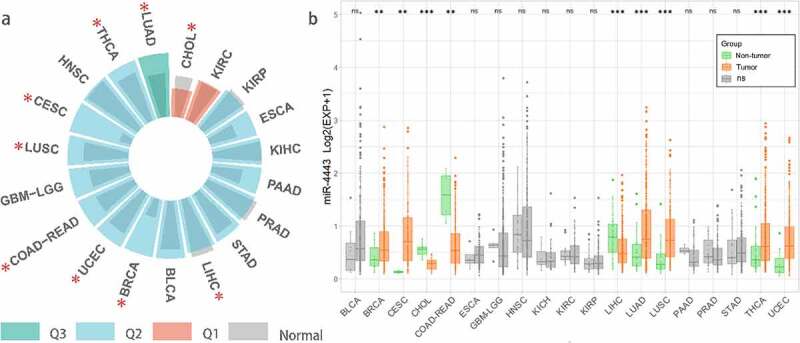
Pan-cancer analysis of miR-4443 using TCGA database.a: * means there is a significant difference (p < 0.05) b: *** means p < 0.001; ** means p < 0.01; * means p < 0.05; ns means no significant difference; BLCA, Bladder urothelial carcinoma; BRCA, Breast invasive carcinoma; CESC, Cervical and endocervical cancers; CHOL, Cholangiocarcinoma; COAD-READ, Colorectal adenocarcinoma; ESCA, Esophageal carcinoma; GBM-LGG, Glioma; HNSC, Head and neck squamous cell carcinoma; KICH, Kidney chromophobe; KIRC, Kidney renal clear cell carcinoma; KIRP, Kidney renal papillary cell carcinoma; LIHC, Liver hepatocellular carcinoma; LUAD, Lung adenocarcinoma; LUSC, Lung squamous cell carcinoma; PAAD, Pancreatic adenocarcinoma; PRAD, Prostate adenocarcinoma; STAD, Stomach adenocarcinoma; THCA, Thyroid carcinoma. UCEC, Uterine corpus endometrial carcinoma;

We downloaded the TCGA (Pan-Cancer) dataset from the UCSC Xena database (https://xenabrowser.net/). Further, we extracted and log2(x + 1) transformed the expression data of miR-4443 in each sample. We eliminated the samples with 0 expression and the cancer species without control samples and finally obtained the expression data of 19 cancer types. In addition, we calculated the median expression of all miRNAs in these 19 cancer types and calculated the rank percentage of miR-4443 among all miRNAs with non-zero expression. As shown in Figure 1a, miR-4443 was highly expressed in LUAD (0.5–0.75 quantile, Q3). miR-4443 was moderately expressed (0.25–0.5 quantile, Q2) in 16 tumors, including THCA, HNSC, CESC, LUSC, GBMLGG, COAD-READ, UCEC, BRCA, BLCA, LICH, STAD, PRAD, PAAD, KICH, ESCA, KIRP. In addition, miR-4443 expression was lower in 2 tumors (KIRC and CHOL) (0–0.25 quantile, Q1).

We calculated differences in miR-4443 expression between normal and tumor samples for 19 cancer types using the unpaired Wilcoxon test of R software (version 4.1.3). As shown in Figure 1b, miR-4443 was significantly up-regulated in 6 tumors (BRCA, CESC, LUAD, LUSC, THCA, and UCEC); miR-4443 was significantly down-regulated in 3 tumors (CHOL, COAD-READ, and LIHC); miR-4443 was not significantly different among 10 tumors, including BLCA, ESCA, GBM-LGG, HNSC, KICH, KIRC, KIRP, PAAD, PRAD, and STAD.

Previous studies found that miR-4443 was highly expressed in BC tumor tissues [6] and NSCLC tumor cells [7], which was consistent with the results in TCGA-BRCA and LUSC/LUAD. In addition, miR-4443 was lowly expressed in HCC tumor tissues [12] and CRC tumor cells [10], which was also validated in TCGA-LIHC and TCGA-COAD/READ.

miR-4443 is lowly expressed in OS tumor cells [11]. Since there were no control samples in the TCGA-SARC database, we could not verify the correlation between miR-4443 and OS in TCGA-SARC. miR-4443 is lowly expressed in GBM tumor cells [13] and highly expressed in mast cells from Glioma patients [8]. However, in TCGA GBM-LGG, the difference of miR-4443 expression between carcinoma and adjacent carcinoma was not obvious. miR-4443 was highly expressed in HNSCC tumor cells (CAL-27 and WSU-HN4) [14], while in TCGA-HNSC miR-4443 was not significantly different between cancerous and paracancerous tissues. By qRT-PCR method, miR-4443 expression was significantly decreased [9] in PTC tumor tissues (n = 30) than in adjacent tissues (n = 30). However, in the TCGA-THCA dataset, the expression of miR-4443 in cancer tissues was higher than that in adjacent tissues. In conclusion, differences in miR-4443 expression may be related to sample types, tumor subtypes, and gene expression detection methods. Furthermore, the association of miR-4443 with cancer risk in different tumors may be related to the presence of tissue-specific upstream regulators such as ceRNAs.

## 2. The molecular mechanisms of iR-4443 affecting cellular behaviors

**Figure 2.**
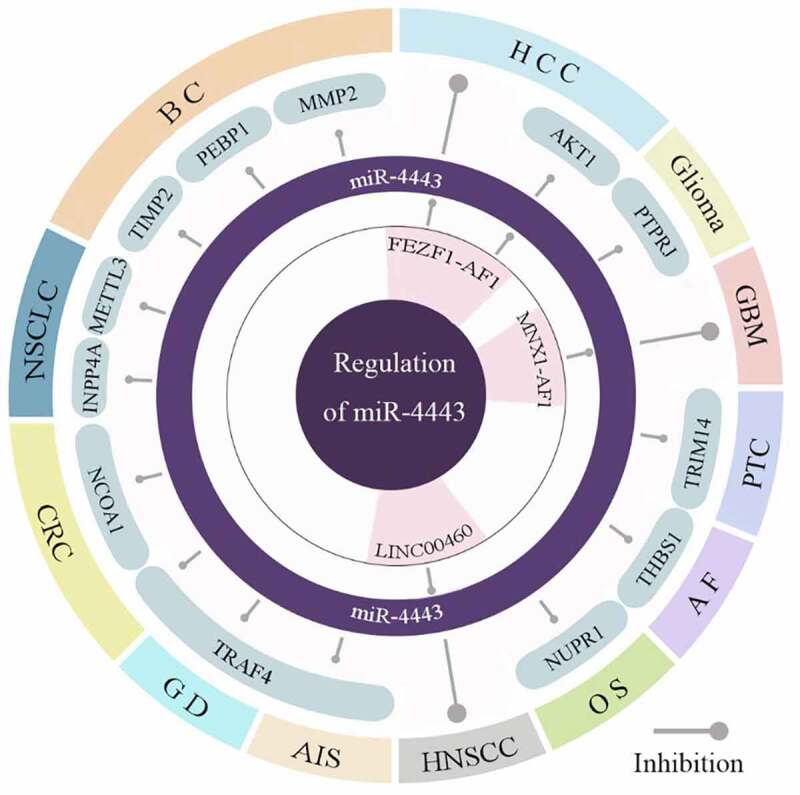
The downstream and upstream genes of miR-4443 in different diseases.miR-4443 inhibits the expression of target genes in various diseases and is involved in three ceRNA regulatory axes.

As shown in Figure 2, miR-4443 not only targets and inhibits 12 target genes, but is also regulated by 3 ceRNAs. The abnormal expression of miR-4443 can lead to dysregulation of downstream gene expression, which in turn affects the abnormality of cellular behaviors, and ultimately leads to the occurrence and development of diseases.

**Figure 3.**
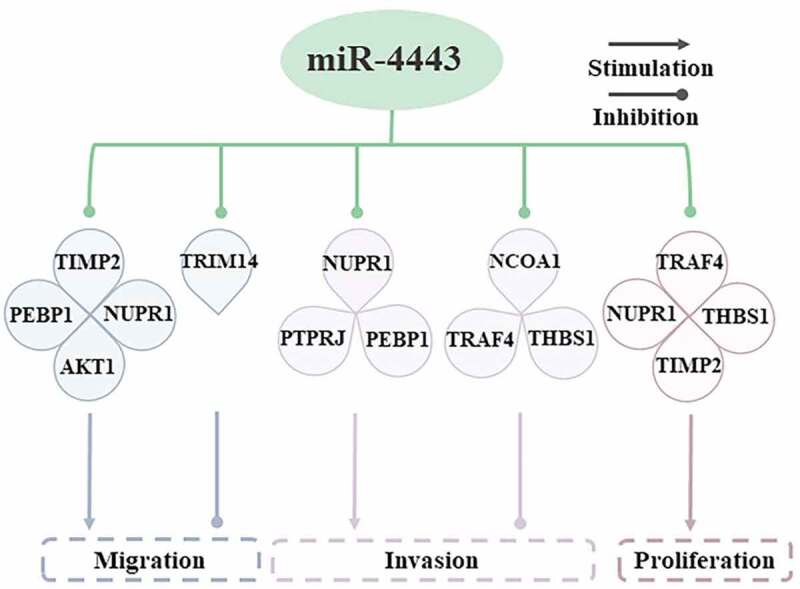
Molecular mechanisms by which miR-4443 affects cell behaviors.miR-4443 affects cell migration, invasion, and proliferation by inhibiting multiple target genes.

Cell migration is the basis for establishing and maintaining the normal tissues of multicellular organisms [19]. The target genes of miR-4443 are closely related to the migration of cancer cells (Figure 3). In breast cancer, miR-4443 inhibits the expression of TIMP metallopeptidase inhibitor 2 (TIMP2) [20] and phosphatidylethanolamine binding protein 1 (PEBP1) [6], thereby promoting the metastasis and invasion of breast cancer. In OS, lncRNA FEZF1-AS1 can sponge miR-4443, thereby promoting the expression of nuclear protein 1 (NUPR1), and leading to the migration, proliferation, and invasion of OS cells [11]. In HCC, lncRNA FEZF1-AS1 can compete to bind miR-4443, thereby up-regulating AKT serine/threonine kinase 1 (AKT1) expression, and ultimately promoting cancer cell metastasis and tumorigenesis [21].

Cell proliferation is an important part of cell growth and differentiation [22]. In different diseases, the effect of miR-4443 on cell proliferation is different. The expression of miR-4443 is upregulated in CD4 + T cells, thereby inhibiting the expression of TNF receptor associated factor 4 (TRAF4) and facilitating the proliferation of CD4 + T cells, and ultimately promoting the occurrence of GD [17]. In OS, the expression level of miR-4443 decreases, which in turn leads to the up-regulation of NUPR1, thereby stimulating the proliferation of OS cells [11]. In the serum of atrial fibrillation (AF) patients, the expression of miR-4443 was significantly reduced, thereby up-regulating the expression level of thrombospondin 1 (THBS1), promoting the proliferation of human cardiac fibroblasts (HCFB), and attenuating cell apoptosis [16].

Invasion of pathological cells to surrounding tissues is a difficult problem for disease treatment [23]. miR-4443 shows two opposite effects in cell invasion. Leptin and insulin treatment of colorectal cancer (CRC) cell line (HCT-116) can elevate the expression of miR-4443, which subsequently leads to down-regulation of nuclear receptor coactivator 1 (NCOA1) and TRAF4, and ultimately hinders the invasion and proliferation of HCT-116 cells [10]. In patients with atrial fibrillation, inhibition of miR-4443 can increase the level of THBS1 expression, thereby promoting the invasion of cardiac fibroblast [16]. In OS, FEZF1-AS1 can sponge miR-4443, thereby up-regulating NUPR1 and promoting OS cell invasion [11]. However, in the BC cell line (MDA-MB-231), the overexpression of miR-4443 suppresses the expression of PEBP1 and promotes the invasion and metastasis of breast cancer cells [6].

In summary, the abnormal expression of miR-4443 in some diseased cells can lead to the dysregulation of downstream gene expression, which in turn affects the abnormal behavior of cells (including migration, proliferation, and invasion), and ultimately leads to the occurrence and development of the disease. In BC and GD, overexpression of miR-4443 promoted cell migration, proliferation, and invasion. However, overexpression of miR-4443 in OS, HCC, AF, and CRC inhibited cell migration, proliferation, and invasion. Due to the small number of samples in miR-4443-related studies, further studies are required to obtain more convincing results.

## 3. The miR-4443 related signaling pathways

There are at least 12 target genes for miR-4443. These include inositol polyphosphate-4-phosphatase type IA (INPP4A) in the Janus kinase 2/signal transducer and activator of transcription 3 (JAK2/STAT3) signaling pathway, THBS1 in the transforming growth factor beta 1 (TGF-β1) signaling pathway, TRAF4 in the nuclear factor kappa B (NF-κB) signaling pathway, and protein tyrosine phosphatase receptor type J (PTPRJ) in the Ras signaling pathway (Figure 5).

The excessive activation of the JAK2/STAT3 signaling pathway is closely related to the occurrence and development of cancer [24]. In non-small cell lung cancer (NSCLC), miR-4443 is significantly related to Epirubicin (EPI) resistance. miR-4443 was highly expressed in the EPI-resistant H1299 cell line. Specifically, miR-4443 promotes the resistance of NSCLC cells to EPI by targeting INPP4A and regulating the activation of the JAK2/STAT3 signaling pathway [7].

TGF-β/Smad signaling promotes the proliferation of fibroblasts and the development of tissue fibrosis [25]. Fibrosis caused by human cardiac fibroblasts plays an important role in the occurrence and development of atrial fibrillation (AF) [26]. In AF patients, thrombospondin-1 (THBS1) can up-regulate the expression of TGF-β1 and smad2/3/4 genes, thereby promoting the proliferation, migration, and invasion of cardiac fibroblasts, and ultimately leading to the differentiation of cardiac fibroblasts. miR-4443 can inhibit THBS1, thereby alleviating fibrosis and AF symptoms [6].

The NF-κB signaling pathway is an important immune-related pathway that can regulate the inflammatory response in body [27]. In AIS patients, TRAF4 increases the phosphorylation level of IκBα in peripheral blood mononuclear cells (PBMCs) and activates the NF-κB signaling pathway. miR-4443 can inhibit the NF-κB signaling pathway by targeting TRAF4, thereby increasing the expression of anti-inflammatory cytokines, inducing immunosuppression, and ultimately increasing the risk of infection after stroke [15]. In addition, miR-4443 inhibits the NF-κB signaling pathway by targeting TRAF4, promotes the transcription of cytokines and chemokines, and up-regulates the proliferation of CD4 + T cells, thereby promoting the risk of GD [17].

The RAS signaling pathway can integrate extracellular signals to control the growth, survival, and differentiation of cell lines. Abnormal activation of the RAS signaling pathway is a major and highly common carcinogenic event [28]. In mast cells, elevated levels of miR-4443 can inhibit PTPRJ, thereby activating the Ras signaling pathway, increasing the release of IL-8 [8], and ultimately inducing angiogenesis and enhancing tumor invasiveness [29].

Accordingly, miR-4443 participates in the regulation of four signaling pathways by regulating downstream genes, thereby affecting the behavior of diseased cells, which is closely related to the expression of cytokines, cell growth and differentiation, and drug resistance.

## 4. The role of miR-4443 in drug resistance

**Figure 4.**
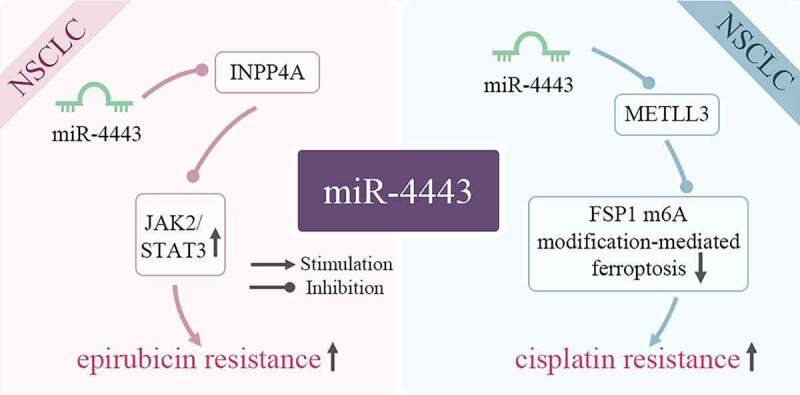
miR-4443 affects cellular drug resistance by inhibiting target genes. In tumor cells, miR-4443 affects the signaling pathway JAK2/STAT3 and normal molecular physiological activities by inhibiting the expression of target genes INPP4A and METLL3, thereby increasing drug resistance.

As shown in Figure 4, miR-4443 is closely related to epirubicin (EPI) and cisplatin resistance of cancer cells. Non-small cell lung cancer (NSCLC) accounts for about 85% of lung cancers, and its incidence is increasing year by year worldwide [30]. Despite significant advances in available therapies for NSCLC, acquired resistance remains a major barrier to NSCLC treatment [31].

**Figure 5.**
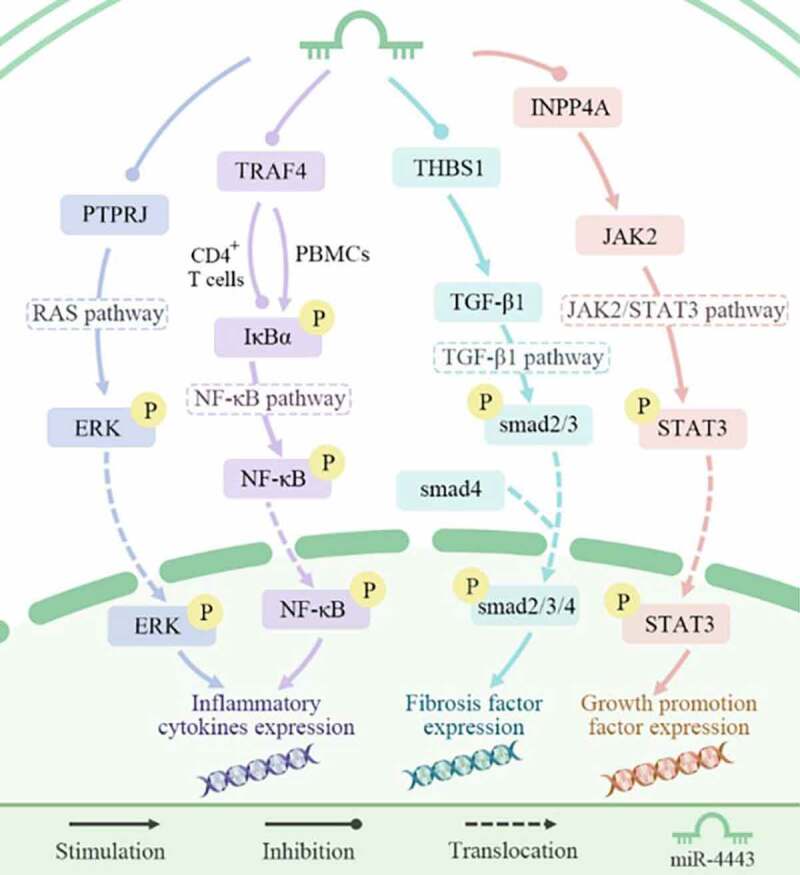
miR-4443 affects biological processes by regulating signaling pathways.miR-4443 regulates the four signaling pathways of JAK2/STAT3, TGF-β1, NF-κB, and Ras by inhibiting four target genes, thereby affecting the expression of inflammatory factors, fibrotic factors, and growth-promoting factors.

Epirubicin (EPI) is an anthracycline antibiotic. It can be used alone or in combination with other drugs to treat advanced non-small cell carcinoma [32]. In NSCLC, miR-4443 is highly expressed in patients insensitive to EPI chemotherapy and EPI-resistant H1299 cells. Overexpression of miR-4443 activates the JAK2/STAT3 pathway by inhibiting the INPP4A gene, and ultimately promotes EPI resistance [7]. In breast cancer, the up-regulation of miR-4443 is also closely related to EPI resistance [33].

Cisplatin is an anti-tumor compound, which can induce ferroptosis and apoptosis of non-small cell lung cancer cells [34]. A previous study has shown that upregulation of miR-4443 can induce A549 resistance to DDP [35]. miR-4443 is significantly up-regulated in exosomes released from cisplatin-resistant NSCLC tumors, resulting in a decrease in the expression of methyltransferase 3 (METTL3) in cisplatin-resistant A549 cells, thereby enhancing the expression of FSP1. Subsequently, the down-regulation of METTL3 also reduces FSP1 m6A modification and FSP1-mediated ferroptosis, and ultimately increases the cisplatin resistance of NSCLC cell lines [36].

Hence, miR-4443 in 2 types of NSCLC cells (A549 and H1299) can induce drug resistance in tumor cells by regulating targets or activating signaling pathways. At present, there is no data to support the link between miR-4443 and drug resistance through the regulation of target genes in other diseases, which requires further research.

Furthermore, target genes regulated by miR-4443 are linked to drug resistance in other diseases. In OC, inhibiting the expression of TIMP2 promoted the proliferation, migration, and cisplatin resistance of A2780 cells [37]. The high expression of THBS1 in GC is not only related to tumor adhesion but also reduces the sensitivity to twelve anticancer drugs such as Oxaliplatin and Tamoxifen [38].

## 5. Discussion

miR-4443 is differentially expressed in 12 diseases. Among them, the expression of miR-4443 is up-regulated in breast cancer(BC), non-small cell lung cancer(NSCLC), glioma, Graves’ disease(GD), and acute ischemic stroke(AIS). The expression of miR-4443 is down-regulated in hepatocellular carcinoma(HCC), osteosarcoma(OS), glioblastoma(GBM), papillary thyroid carcinoma(PTC), head and neck squamous cell carcinoma(HNSCC), colorectal cancer(CRC), and atrial fibrillation(AF).

In addition, miR-4443 also plays an important role in human non-cancer diseases (Figure 2). The low expression of miR-4443 in AF upregulates the expression of its target gene THBS1, which promotes HCFB proliferation and leads to AF. Upregulation of miR-4443 in acute ischemic stroke (AIS) increases its risk. High expression of miR-4443 in Graves’ disease (GD) promotes the transcription of cytokines and increases the risk of GD. miR-4443 is highly expressed in AIS and increases the risk of post-stroke infection by inhibiting TRAF4.

The expression of miR-4443 is regulated by three ceRNAs. Although long non-coding RNAs (lncRNAs) do not encode proteins, they can regulate the expression of downstream target protein-coding genes through a variety of mechanisms. Especially for cancer, dysregulation of lncRNAs is associated with various malignant phenotypes and leads to cancer progression and metastasis [39]. Therefore, the differential expression of miR-4443 in different diseases may be related to the regulation of ceRNA. The study found that compared with the normal control group, miR-4443 was highly expressed in glioma tissue and serum [40], showing its great potential in diagnosis.

In addition, our work also shows that miR-4443 regulates cell migration, proliferation, and invasion by down-regulating the expression level of its target genes, and participates in the regulation of signaling pathways such as JAK2/STA T3, TGF-β1, NF-κB, and Ras. The aberrent expression of miR-4443 is also closely related to the resistance of EPI and cisplatin.

miR-4443 is closely related to the prognosis of various tumors. In hepatocellular carcinoma (HCC), patients with low miR-4443 expression had significantly reduced overall survival [12]. In breast cancer (BC) [33] and lung adenocarcinoma (LUAD) [41], high expression of miR-4443 resulted in decreased overall survival. Upstream regulators of miR-4443 include lncRNA FEZF1-AS1 [12] and lncRNA ENST0000630242 [41]. The overall survival rate of HCC patients with high expression of lncRNA FEZF1-AS1 was significantly decreased [12], while the overall survival rate of LUAD patients with high expression of lncRNA ENST0000630242 was increased [41]. Therefore, the difference in the relationship between miR-4443 and the prognosis of patients with different cancers may be related to the existence of different upstream regulators of miR-4443.Due to the limitation of sample type and number of samples, the role of miR-4443 in diagnosis and prognosis needs to be further verified.

Therefore, there are still many shortcomings in the related research of miR-4443. First of all, the sample sizes in the current studies are small, and their results need to be verified in larger samples and other populations. Secondly, the molecular mechanisms of miR-4443 in diseases are still not fully understood, and more in-depth research is needed in the future. Finally, the diagnostic and prognostic value of miR-4443 remains to be further evaluated.

## 6. Conclusions

As an important regulatory molecule, miR-4443 is closely related to the development of many diseases, especially cancer. This work provides an overview of the aberrant expression of miR-4443 in cancer and non-cancer diseases, revealing its molecular mechanisms in cellular behavior. In addition, this study elucidates the regulatory role of miR-4443 in signaling pathways and the drug resistance induced by miR-4443, which provides potential clues and directions for the follow-up research of miR-4443.

## Data Availability

All data generated or analyzed during this study are included in the article.
